# Deep sequencing on a genome-wide scale reveals diverse stage-specific microRNAs in cambium during dormancy-release induced by chilling in poplar

**DOI:** 10.1186/s12870-014-0267-6

**Published:** 2014-10-01

**Authors:** Qi Ding, Jun Zeng, Xin-Qiang He

**Affiliations:** College of Life Sciences, Peking University, Beijing, 100871 China

**Keywords:** Cambium, Chilling, Ecodormancy, Endodormancy, MiRNAs, Poplar

## Abstract

**Background:**

Trees in temperate zones show periodicity by alternating active and dormant states to adapt to environmental conditions. Although phytohormones and transcriptional regulation were found to be involved in growth cessation and dormancy transition, little is known about the mechanisms of the dormancy-active growth transition, especially dormancy maintenance and release. Small RNAs are a group of short non-coding RNAs regulating gene expressions at the post-transcriptional level during plant development and the responses to environmental stress. No report on the expression profiling of small RNAs in the cambial meristem during the dormancy-active growth transition has been reported to date.

**Results:**

Three small RNA libraries from the cambium of poplar, representing endodormancy induced by short day conditions, ecodormancy induced by chilling and active growth induced by long day conditions, respectively, were generated and sequenced by Illumina high-throughput sequencing technology. This yielded 123 known microRNAs (miRNAs) with significant expression changes, which included developmental-, phytohormone- and stress-related miRNAs. Interestingly, miR156 and miR172 showed opposite expression patterns in the cambial dormancy-active growth transition. Additionally, miR160, which is involved in the auxin signaling pathway, was expressed specifically during endodormancy release by chilling. Furthermore, 275 novel miRNAs expressed in the cambial zone were identified, and 34 of them had high detection frequencies and unique expression patterns. Finally, the target genes of these novel miRNAs were predicted and some were validated experimentally by 5′RACE.

**Conclusions:**

Our results provided a comprehensive analysis of small RNAs in the cambial meristem during dormancy-release at the genome-wide level and novel evidence of miRNAs involved in the regulation of this biological process.

**Electronic supplementary material:**

The online version of this article (doi:10.1186/s12870-014-0267-6) contains supplementary material, which is available to authorized users.

## Background

Trees in temperate zones show periodicity by alternating active and dormant states to adapt to natural conditions, such as light, temperature and drought. Growth arrest is the first step of plant dormancy, followed by the dormant state, which can be divided into two stages: ecodormancy (or quiescence) and endodormancy (or rest). In ecodormancy, plants can restore active growth upon exposure to growth-promoting conditions, while plants in endodormancy cannot [1].

Photoperiod has been known to govern the growth cessation of many trees in temperate climates. Plants sense changes in the photoperiod through the leaves and send a graft-transmitted message to the terminal, so that the terminal initiates dormancy [1,2]. The identification of poplar *FLOWERING LOCUS T* (*FT*) and *CONSTANS* (*CO*) as mediators of growth cessation induced by the short day (SD) photoperiod was a significant breakthrough in the study of dormancy transition regulations [3]. *Like-AP1* (*LAP1*), a poplar ortholog of the *Arabidopsis* gene *APETALA1* (*AP1*), mediates the photoperiodic control of seasonal growth cessation downstream of *CO/FT* [4]. Another environmental factor that controls dormancy transition is temperature. Low temperature plays an important role in inducing growth cessation and dormancy [5,6]. However, a continuous chilling must occur to release endodormancy and switch to ecodormancy, and then warm temperatures in the spring subsequently reinitiate growth [6].

Environmental factors are thought to regulate the precise annual cycle’s time course by modulating phytohormone levels or altering the sensitivity of the cells to phytohormones. So far, gibberellins and auxin are widely recognized as the most important phytohormones involved in the dormancy transition [7-11]. Applications of exogenous gibberellins could cause the dormant poplar buds to sprout without chilling, and it is shown that a low temperature could alter the expression of key regulators in the gibberellin signal pathway [11,12]. Auxin has crucial roles in cambial cell division, which makes it very important in the dormancy-active growth transition [13,14]. A recent study shows that the induction of cambial growth cessation and dormancy involves changes in auxin responses rather than auxin content [7]. Another phytohormone that may participate in the dormancy-active growth regulation is abscisic acid (ABA), which peaks in poplar apical buds after growth cessation and before bud set [15-17].

So far, the studies on mechanisms of the cambial dormancy-active growth cycle have mainly focused on hormonal [9,10,12,15] and transcriptional regulation [16-18]. The switch between plant dormancy and active growth is a complex biological phenomenon that involves a large number of genes and many metabolic processes, as well as the interactions of a variety of hormones. Multiple levels of control networks are involved in such complex biological events, in addition to transcriptional and protein regulation.

Small RNAs (sRNAs), short (~21 nt) non-coding RNAs, are important regulators of gene expression at the post-transcriptional level during plant development and response to environmental stress [19]. sRNAs, in particular microRNAs (miRNAs), have been studied extensively in poplar, including genome-wide profiling of sRNAs and miRNAs [20,21] and stress responses to drought [22,23], salt [24], cold [25] and pathogens [26]. In addition, some miRNAs have been found to be of great importance in tree development. For instance, miR166 is reported to be involved in vascular tissue development [27,28] and may be related to the cambial active period [29]. MiR156 and miR172, which is well studied in *Arabidopsis*, appear not only to control flowering and the timing of sensitivity in response to vernalization, but also vegetative phase changes in trees [30-35]. Although comprehensive work has been done to describe miRNAs in trees during various cellular processes, there is no report on the expression profiling of miRNAs in the cambial meristem during the dormancy-active growth transition and little is known about the regulation of miRNAs in the process. In this paper, we present a deep sequencing profile on a genome-wide scale that reveals stage-specific miRNAs in the cambial zone during this process. Millions of sRNA reads were obtained, and after further analysis, we found 123 known miRNAs, including developmental-, phytohormone- and stress-related miRNAs, which showed significant expression-level changes during dormancy-release by chilling. Furthermore, 275 novel miRNAs expressed in the cambium zone were identified, and 34 of them had high detection frequencies and unique expression patterns. The target genes of these novel miRNAs were predicted and some of them were validated. Our results revealed the expression changes of miRNAs in cambium dormancy-release by chilling in poplar, and provided evidence of miRNA involvement in the regulation of the dormancy-active growth transition of trees.

## Results

### Dormancy-active growth transition induced by photoperiod and chilling in poplar

**Figure 1:**
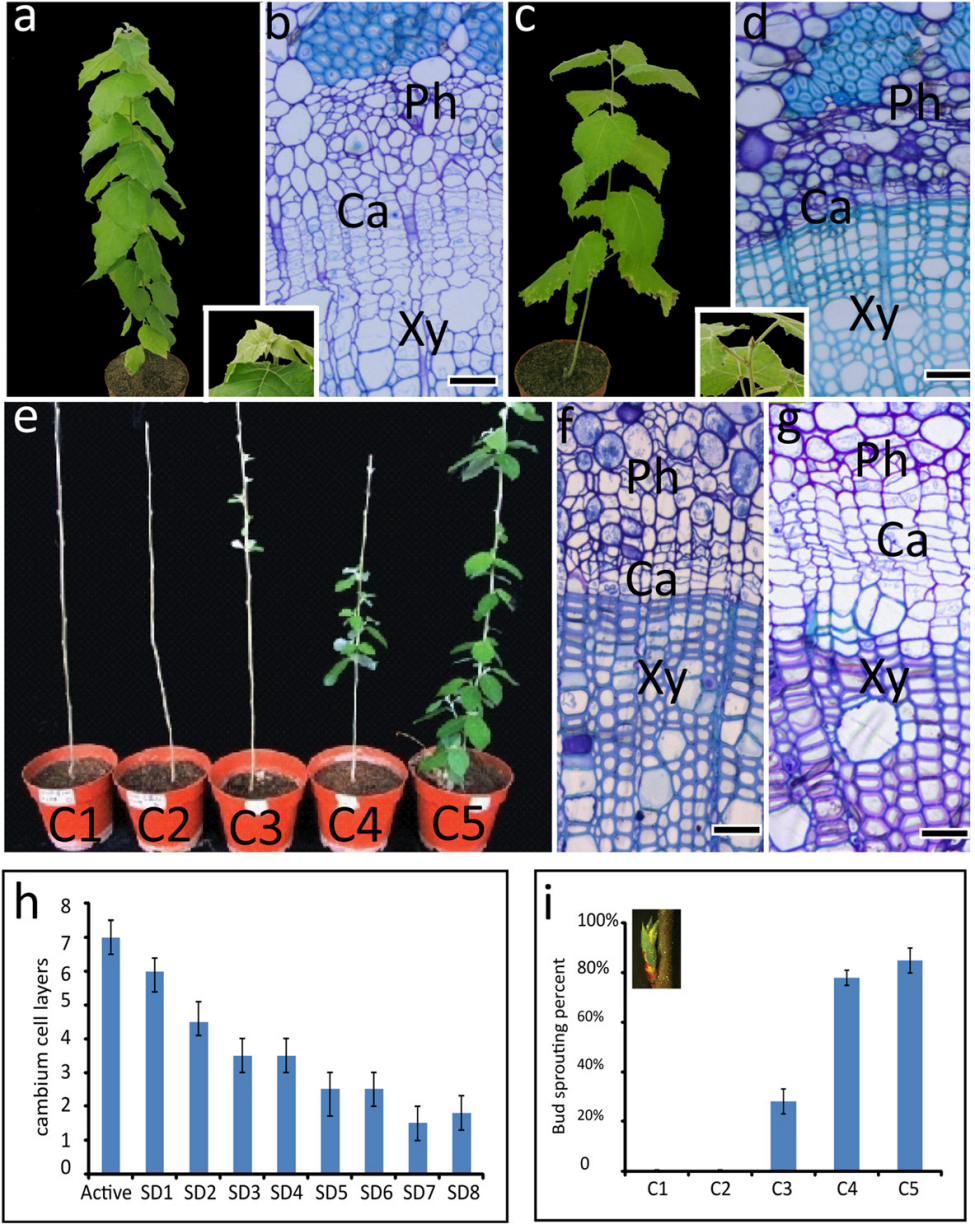
**The dormancy-active growth transition induced by photoperiod and chilling in poplar. a-b**: a poplar tree in active growth **(a)** and its stem cross section showing the anatomical features of active cambial cells **(b)**; Magnification of the stem apex was shown in the insert picture between **(a)** and **(b)**; **c-d**: the endodormancy state induced by SD treatment for 8 weeks **(c)** and the stem cross section showing the anatomical features of cambial cells in endodormancy **(d)**; Magnification of a dormant apical bud was shown in the insert picture between **(c)** and **(d)**. **e**: the trees growing in LD for 3 weeks after chilling treatment of 1–5 weeks (C1 to C5), showing the effects of different chilling treatments on the dormancy-release. **f-g**: the cross sections of stem C1 **(f)** and C5 **(g)**; **h**: a statistical chart for cambial cell layers through a SD treatment for 8 weeks. **i**: a statistical chart of bud sprouting percent for the dormancy-release after chilling treatment for 5 weeks, A bud sprouting was shown in the insert figure in **(i)**. SD1-8: short day treatment for 1–8 weeks; LD: long day; C1-C5: chilling treatment for 1–5 weeks; Ph: phloem; Ca: cambium; Xy: xylem; bars = 100 μm.

The induction of dormancy and resumption of growth in poplar were constructed according to Espinosa-Ruiz *et al*. [36] with some modifications. After 8 weeks of the short day (SD) treatment of 8 h light/16 h dark, the tree growth was arrested. Dormant apical buds formed (Figure 1a, c) and the layers of cambial cells (Figure 1b, d) decreased from 6–8 to 1–2 (Figure 1h). Although trees were transferred to the long day (LD) condition of 16 h light/8 h dark at this time, they would not resume growth, indicating their endodormant state. To release endodormancy, the 8-week SD-treated trees were exposed to chilling temperatures of 4°C. Only trees exposed to a chilling treatment for at least 4 weeks could resume growth, which was shown in bud burst, and cambial cell division and differentiation, when they were transferred to LD conditions at 25°C for 3 weeks (Figure 1e, f, g, i). The results indicated that the endodormant state was released and that the trees had shifted to the ecodormant state after 5 weeks of the chilling treatment. Then, the active growth state was induced by 3 weeks of the LD condition at room temperature.

### Deep sequencing of sRNAs in cambium during dormancy-release in poplar

To investigate the miRNA component of sRNAs and the changes of miRNAs in cambial meristem during dormancy-release in poplar, three sRNA libraries from the cambium of poplar, representing endodormancy with an 8-week SD treatment (SD8), ecodormancy with a 5-week chilling treatment (C5) and active growth under a 3-week LD condition (LD3) after chilling, respectively, were generated and sequenced by Illumina high-throughput sequencing technology. Raw read totals of 16,688,990, 21,379,082 and 15,942,869 from SD8, C5 and LD3, respectively, were acquired. After removal of low-quality sequences, adapter sequences, polyA sequences, sequences smaller than 18 nucleotides and other artifacts, we obtained 16,339,437, 20,887,480 and 15,649,238 high-quality 18 to 30 nt sRNA clean reads in SD8, C5 and LD3, respectively, for further analysis (Table 1).

**Table 1:** **Statistics of sRNAs in cambium during dormancy-release in poplar**

**Type**	**Endodormancy(SD8)**		**Ecodormancy(C5)**		**Activity(LD3)**	
	**Count**	**Percent**	**Count**	**Percent**	**Count**	**Percent**
total_reads	16688990		21376082		15942869	
high_quality	16599916	100%	21259764	100%	15865743	100%
3'adapter_null	8986	0.05%	10757	0.05%	8894	0.06%
insert_null	2574	0.02%	2553	0.01%	2107	0.01%
5'adapter_contaminants	28453	0.17%	18969	0.09%	18278	0.12%
smaller_than_18nt	220169	1.33%	339653	1.60%	186610	1.18%
polyA	297	0.00%	352	0.00%	616	0.00%
clean_reads	16339437	98.43%	20887480	98.25%	15649238	98.64%

**Figure 2:**
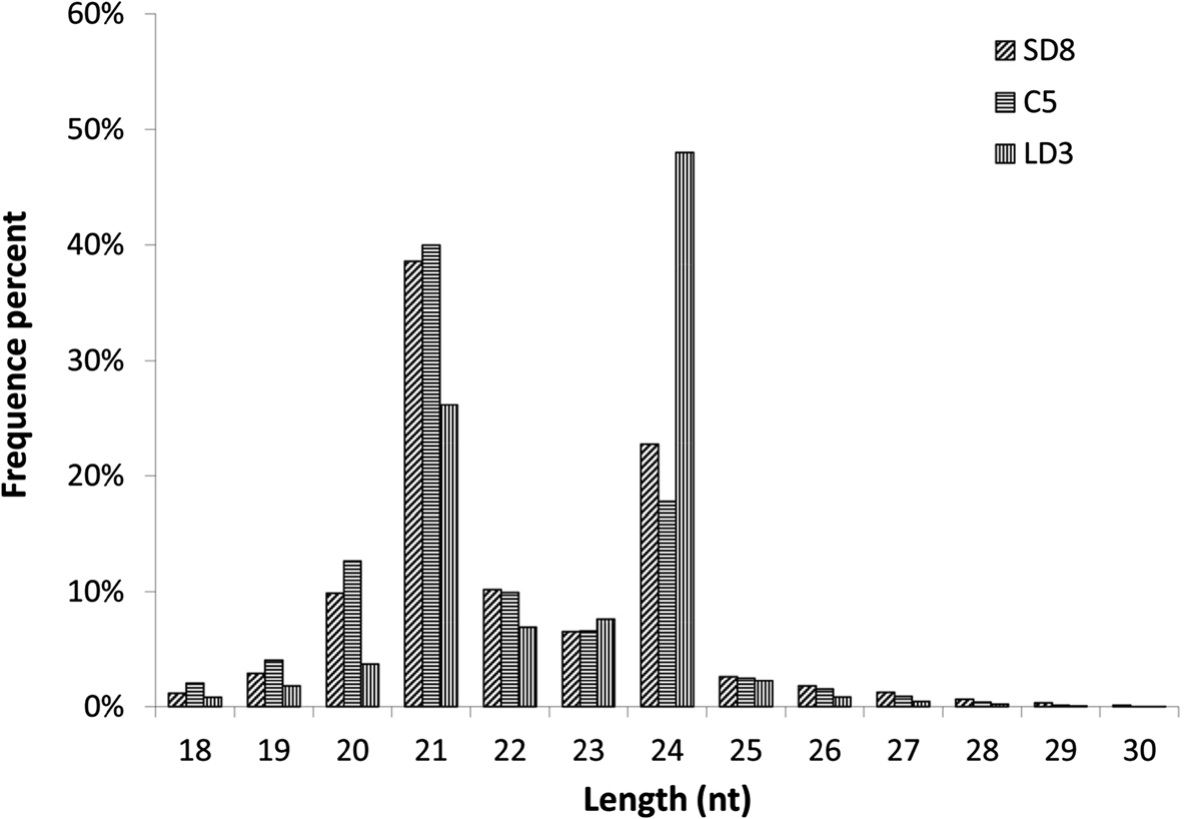
**Size distribution of unique sRNAs identified from the cambium during dormancy-release in poplar.** SD8: short day treatment for 8 weeks; C5: chilling treatment for 5 weeks; LD3: long day treatment for 3 weeks.

Among the 18 to 30 nt sRNA clean reads from sequencing, the majority of them (65%) were in the range of 20 to 24 nt in length, with sequences of 21 nt or 24 nt representing the most abundant classes in each library (Figure 2). The major component of the sRNAs in SD8 and C5 was 21 nt long; however, the proportion of 24 nt sRNAs peaked in LD3 (Figure 2).

sRNA libraries generated by sequencing were complex in composition, including miRNAs, siRNAs, rRNAs, tRNAs, small nuclear RNAs (snRNAs) and small nucleolar RNAs (snoRNAs). To annotate the sRNAs, we first mapped the sRNAs of 18 to 30 nt to the *Populus trichocarpa* genome (www.phytozome.net) using SOAP software (http://soap.genomics.org.cn), and then characterized each kind of sRNA by aligning them to certain databases. Known miRNAs were identified by alignment to sequences in miRBase 20.0 with no mismatches. Meanwhile, the Rfam9.1, NCBI and GenBank databases were employed to annotate the other kinds of sRNAs, including scRNAs, rRNAs, tRNAs, snRNAs and snoRNAs. The repeats that represented the sRNAs positioned at repeat loci were identified using Tag2repeat software. In addition, there were possibly degraded species of mRNAs in the sRNA libraries, which were determined through intron/exon alignment. The remaining unannotated sRNAs were candidates for predicting novel miRNAs and potential miRNA seeds edit. As a result, 573,822, 618,526 and 844,787 unique sRNAs in SD8, C5 and LD3, respectively, were mapped perfectly to the genome, and the proportions for each kind of sRNA were listed in Table 2. Interestingly, the miRNAs represented 22.68% and 24.92% of the total sRNA reads in SD8 and C5, respectively, but only 13.45% in LD3. There were ~200 more unique miRNAs in LD3 than in both endodormancy and ecodormancy (Table 2), indicating that the miRNA population in active cambium was more diversified, which may be due to the complex cellular processes associated with active growth.

**Table 2:** **Annotations of sRNAs perfectly matching poplar genome**

**Type**	**Endodormancy(SD8)**				**Ecodormancy(C5)**				**Activity(LD3)**			
	**Unique**	**Percent**	**Total**	**Percent**	**Unique**	**Percent**	**Total**	**Percent**	**Unique**	**Percent**	**Total**	**Percent**
**Total**	3487733	100%	16339437	100%	3470605	100%	20887480	100%	5854401	100%	15649238	100%
**exon_antisense**	34899	1.00%	105910	0.65%	37745	1.09%	121410	0.58%	42031	0.72%	105942	0.68%
**exon_sense**	77352	2.22%	313316	1.92%	112715	3.25%	379211	1.82%	86849	1.48%	282268	1.80%
**intron_antisense**	8997	0.26%	26209	0.16%	9071	0.26%	27332	0.13%	14350	0.25%	45547	0.29%
**intron_sense**	15080	0.43%	107476	0.66%	17907	0.52%	124816	0.60%	21054	0.36%	112381	0.72%
**miRNA**	1479	0.04%	3705237	22.68%	1496	0.04%	5205158	24.92%	1698	0.03%	2105039	13.45%
**rRNA**	135819	3.89%	3749479	22.95%	143627	4.14%	5370005	25.71%	77931	1.33%	1229193	7.85%
**repeat**	194614	5.58%	474134	2.90%	192856	5.56%	505272	2.42%	339626	5.80%	828424	5.29%
**snRNA**	4644	0.13%	17338	0.11%	5485	0.16%	23259	0.11%	3739	0.06%	11249	0.07%
**snoRNA**	3171	0.09%	13771	0.08%	3665	0.11%	17953	0.09%	2610	0.04%	9582	0.06%
**tRNA**	46851	1.34%	721491	4.42%	47666	1.37%	1388134	6.65%	59169	1.01%	468764	3.00%
**Unannotated**	2964827	85.01%	7105076	43.48%	2898372	83.51%	7724930	36.98%	5205344	88.91%	10450849	66.78%

### Identification and expression profiles of known miRNAs in cambial meristem during dormancy-release in poplar

Known miRNAs in the cambium of poplar were annotated by alignment to the sequences in the available poplar miRNA database. As a result, we identified 182 mature miRNA, two miRNA-5p, two miRNA-3p and 183 pre-miRNAs in SD8, 176 mature miRNA, two miRNA-5p, two miRNA-3p and 177 pre-miRNAs in C5, and 175 mature miRNA, two miRNA-5p, two miRNA-3p and 176 pre-miRNAs in LD3. All the mature miRNAs identified belonged to 33 conserved and non-conserved miRNA families, of which 123 known miRNAs in 26 miRNA families showed significant expression-level changes during this process (Additional file 1: Table S1).

To elucidate the potential regulatory roles of these miRNAs in the dormancy-active growth transition, we analyzed the miRNAs with unique expression patterns during the process, which were mainly involved in plant development and stress response, as well as the plant hormone signal pathway.

**Figure 3:**
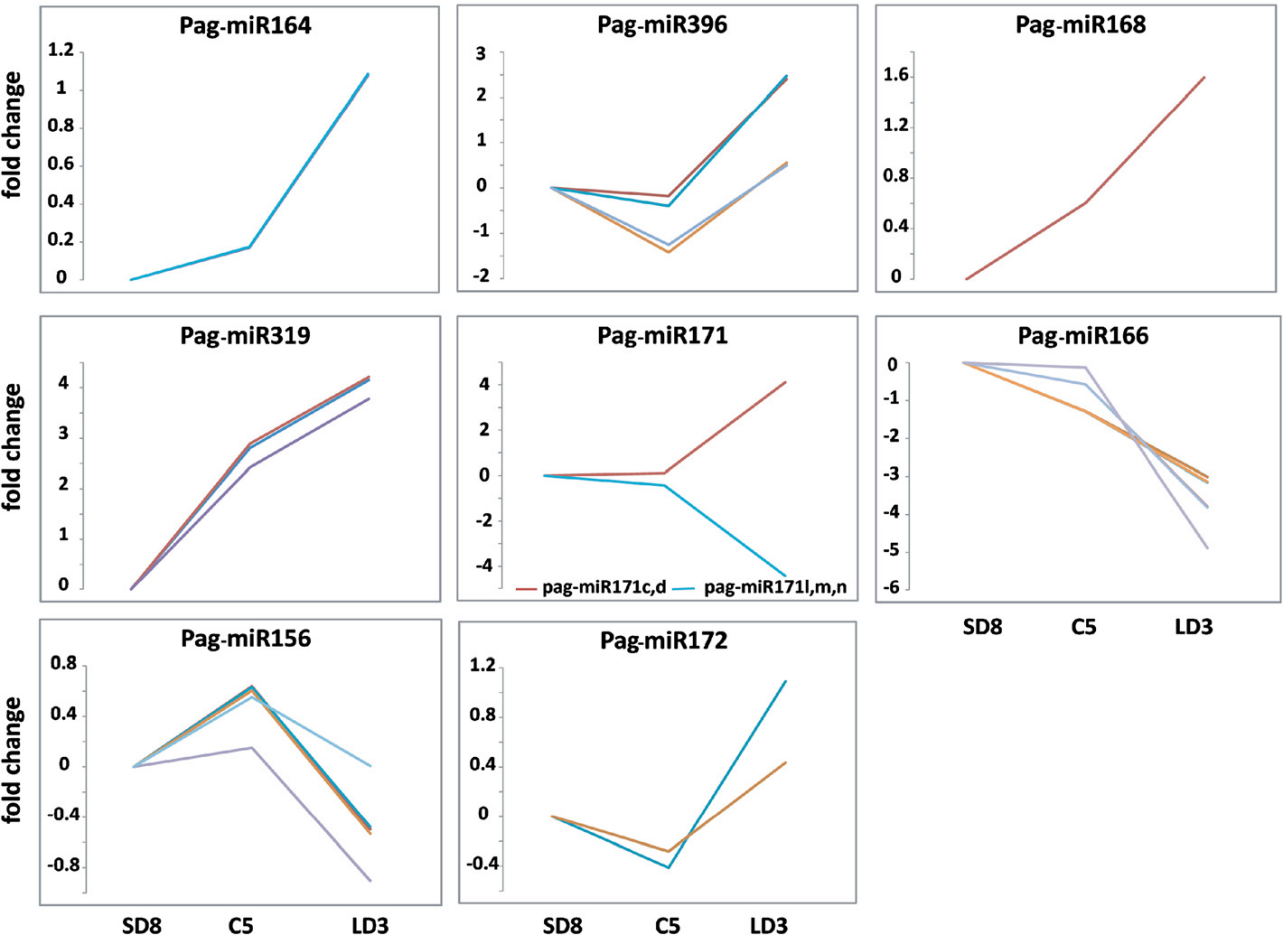
**The fold change of development-related known miRNAs during dormancy-release in poplar.** The y-axis represented the fold change (log2 value) of normalized miRNA counts. SD8 was arbitrarily set to be the control of C5 and LD3. SD8: short day treatment for 8 weeks; C5: chilling treatment for 5 weeks; LD3: long day treatment for 3 weeks.

In our dataset, eight differentially expressed known development-related miRNA families were detected, including miR164, miR396, miR168, miR319, miR171, miR166, miR156 and miR172 (Figure 3). These miRNAs functioned in cell proliferation (miR164, miR396 and miR319) [37-40], vascular development (miR166) [41] and miRNA biogenesis (miR168) [42]. Most of these development-related miRNAs were enriched in the active growth stage. miR319 increased dramatically from SD8 to C5 and continued at a high expression level from C5 to LD3, suggesting that the expression of miR319 may be affected by the chilling treatment. However, miR164, miR168 and miR396 showed no obvious, or only slight, changes from SD8 to C5, but increased in LD3. Intriguingly, unlike other development-related miRNAs, miR166 was enriched in SD8 and C5, and was nearly undetectable in LD3. The members of the miR171 family showed different expression patterns; some of them were highly expressed, while some were repressed during the active growth, indicating that members from one family could have distinct functions in this process.

miR156 and miR172 are well known for controlling the meristem cell fate transition in maize [30-33], *Arabidopsis* [34] and the vegetative phase change in trees [35]. In our study, 10 miRNA members of the miR156/157 family and six miRNA members of the miR172 family were identified. Intriguingly, miR156 was highly expressed in SD8 and C5, and then decreased in LD3, while miR172 had the opposite expression pattern (Figure 3), which showed a similar expression pattern during the vegetative phase change in trees [35].

**Figure 4:**
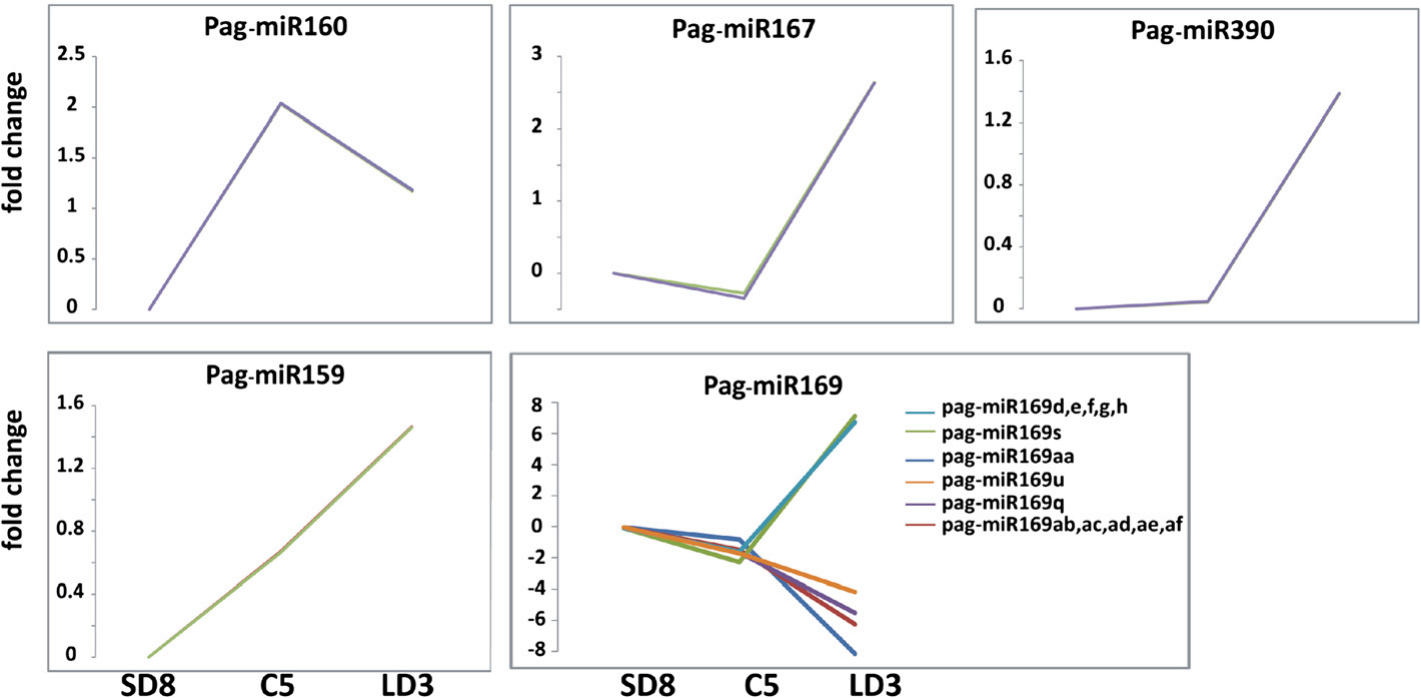
**The fold change of phytohormone-related known miRNAs during dormancy-release in poplar.** The y-axis represented the fold change (log2 value) of normalized miRNA counts. SD8 was arbitrarily set to be the control of C5 and LD3. SD8: short day treatment for 8 weeks; C5: chilling treatment for 5 weeks; LD3: long day treatment for 3 weeks.

To investigate miRNAs involved in the process through the plant hormone pathway, the dynamic expression levels of hormone-related miRNAs were analyzed. Auxin signaling-related miR160, miR167 and miR390 had distinct differential expression patterns in the dormancy-active growth transition (Figure 4). The expression of miR160 peaked in C5, which was the phase sensitive to auxin treatment in dormancy. The unique enrichment in ecodormancy suggested miR160 had an important role in the transition from endodormancy to ecodormancy. Unlike miR160, miR167 and miR390 maintained low expression levels from SD8 to C5, and then increased dramatically from C5 to LD3 (Figure 4), indicating that miR167 and miR390 may function in the auxin pathway during active growth.

Several members of the miR169 family, whose target genes participated in ABA resistance [43,44], were identified in the cambium during the dormancy-active growth transition. The expression levels of all the miR169 members stayed basically unchanged during SD8 and C5, while in LD3, they displayed opposite trends (Figure 4). These findings showed that members of the miR169 family had different functions in this process.

The miR159 family repressed the conserved *GAMYB-like* genes that have been implicated in gibberellin (GA) signaling in anthers and germinating seeds [45]. We found that miR159 was highly expressed in C5 and kept rising in LD3 (Figure 4). The GA signal had already been proven to be a key factor during dormancy-release in poplar [11,12]. The expression change of miR159 raised the possibility that it may be involved in this mechanism through the GA signal pathway.

**Figure 5:**
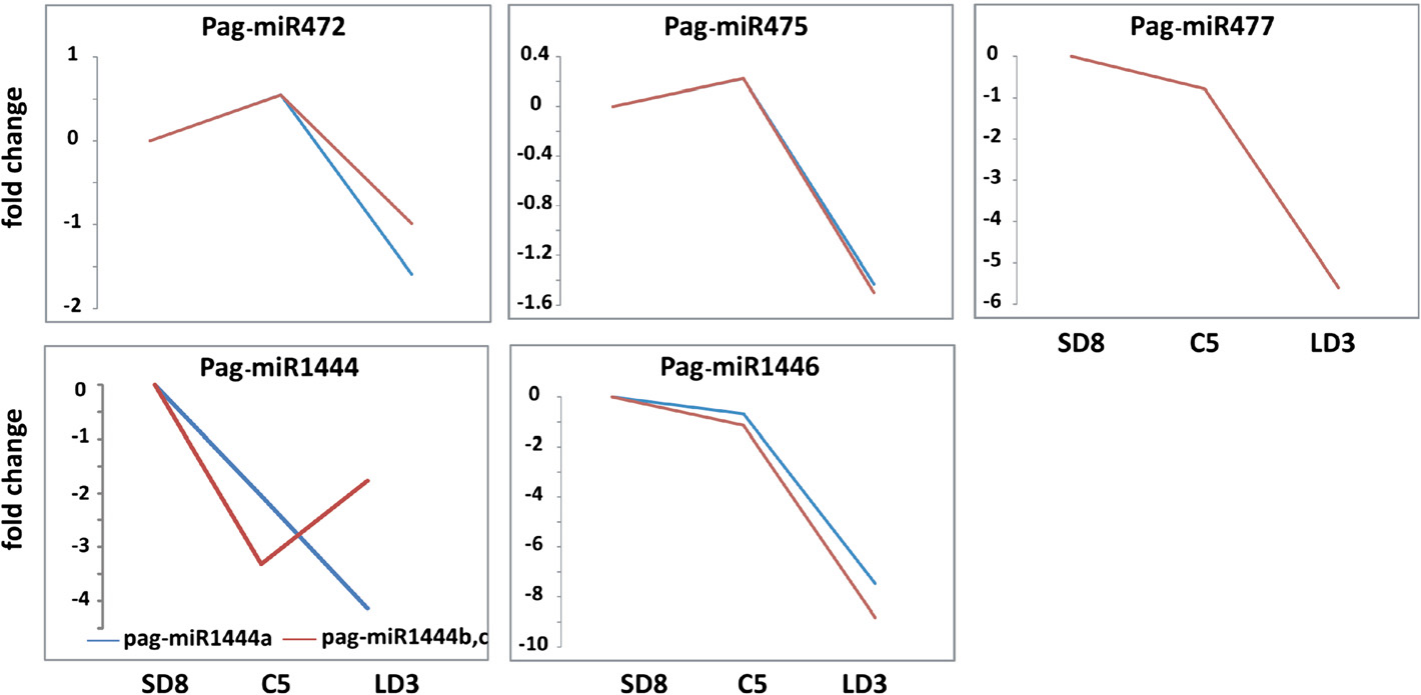
**The fold change of tolerance-related known miRNAs in during dormancy-release in poplar.** The y-axis represented the fold change (log2 value) of normalized miRNA counts. SD8 was arbitrarily set to be the control of C5 and LD3. SD8: short day treatment for 8 weeks; C5: chilling treatment for 5 weeks; LD3: long day treatment for 3 weeks.

Lu *et al*. identified 68 stress tolerance-related miRNAs in poplar [46]. Among them, miR472, miR475, miR477, miR1444 and miR1446 were found to show differential expression levels in this study (Figure 5). The abundance of miR1444 greatly dropped, while those of the others changed slightly from SD8 to C5. All five of these miRNAs had lower expression levels in LD3 (Figure 5).

### Identification and expression profiles of novel miRNAs

The Mireap software was employed to screen novel miRNAs from candidates by exploring not only the secondary hairpin structure, but also the Dicer cleavage site and the minimum folding free energy (MFE). According to the analyses, more than 80% of the candidate novel miRNAs in SD8 and C5 began with a 5′uridine, which was a conserved feature of miRNAs recognized by the ARGONAUTE 1 (AGO1) protein [47]. However, in LD3, this ratio was reduced to ~50%. To ensure the authenticity of novel miRNAs, several conditions must be satisfied: the lengths of the mature candidate novel miRNAs varied from 20 to 23 nucleotides, the number of reads was greater than five, and all the unique sequences were identified in at least one library. As a result, we obtained 128, 110 and 147 unique sequences in SD8, C5 and LD3, respectively. After removing the redundancy, 275 novel miRNAs were identified in the three libraries. The average MFE value of novel miRNAs in each library was −55.37 ± 21.50 kcal/mol in SD8, −50.39 ± 19.03 kcal/mol in C5 and −53.75 ± 21.72 kcal/mol in LD3. Most of these novel miRNAs were only expressed at a specific stage, and only a few of them were expressed in two of three libraries. We found 55 specific unique sequences in SD8, 50 in C5 and 92 in LD3 (Additional file 2: Table S2). Most of these novel miRNAs had low detection frequencies in all three libraries. Here, we listed the miRNAs whose detection frequencies were greater than 20 in at least one library and that had marked expression-level changes (Table 3).

**Table 3:** **Novel miRNAs having obviously expression level changes during the dormancy-activity transition**

**miRNA**	**Mature sequence**	**Location**		**Count**	
			**SD8**	**C5**	**LD3**
0A-m0034_5p	TCATGGTTGTTGTGGACAGAT	scaffold_14:916309:916476	38	0	24
0A-m0035_5p	GGGTGTTTGGAAGTGTGGTAGC	scaffold_14:1335805:1336149	12	0	36
0A-m0134_3p	TGTTTGGAAGTGTGGTTATGGTT	scaffold_6:9337050:9337391	27	0	78
0A-m0013_3p	TGTTTGGAAGTGTGGTAGTGGTT	scaffold_11:18019149:18019329	40	0	168
0A-m0051_5p	TAATCTGCATCCTGAGGTTTG	scaffold_16:8456146:8456227	7	8	178
0A-m0066_3p	AGAGGGTGTTTGAGAGTGTGGTT	scaffold_18:12990001:12990327	6	12	68
0A-m0062_3p	TGGCTAAGCTGACAGGCTCTTC	scaffold_17:7526151:7526481	11	13	95
0A-m0050_3p	AACAAGTGCATGAGACTCGGA	scaffold_16:3063731:3063834	25	28	160
0A-m0146_5p	TTCAGATCAGTAGATAGCATG	scaffold_8:207755:207852	66	48	106
0A-m0078_5p	TATTATTGTAAACAAGCTGAC	scaffold_1:38115909:38116118	56	115	8
0A-m0045_5p	GCCGTCTTAGCTCAGCTGGTA	scaffold_15:14906320:14906473	99	141	23
0A-m0007_3p	TTGCCGACCCCACCCATGCCAA	scaffold_10:12814698:12814812	63	177	32
0A-m0004_5p	TTTAATTTCCTCCAATATCTCA	scaffold_10:20020286:20020432	90	212	15
0A-m0084_5p	TCGTAATGCTTCATTCTCACAA	scaffold_1:22901409:22901514	135	226	13
0A-m0077_5p	TTAAATGATGACATGGACACC	scaffold_1:35822737:35822929	386	424	80
0A-m0108_5p	GCTGGAGTAGCTCAGTTGGTT	scaffold_4:16304187:16304406	399	760	151
0A-m0114_3p*	TTGTACACAGAATAGGTGAAAT	scaffold_5:1237647:1237753	1718	1991	863
0A-m0149_5p*	CATCTTGATCAATGGCCATTG	scaffold_8:14223464:14223609	2214	1857	931
0A-m0057_5p#	TAACATCTTGATCAATGGCCA	scaffold_17:1876674:1876823	2239	1884	954
5A-m0010_5p	TAATATTTTGATCGGATCTCGG	scaffold_11:8804382:8804487	0	81	0
5A-m0081_3p	TCTTTAGACAGGCTAGAATCG	scaffold_2:11607389:11607598	0	192	0
5A-m0104_3p*#	TTACCAATACCTCTCATGCCAA	scaffold_5:11901572:11901665	0	2089	0
5A-m0027_3p*	TTGAGGAGAATGAGCAAGGGG	scaffold_14:5359777:5359978	0	328	68
A-m0003-5p#	TGGGCGCGTTGGGGCTGCTTAT	scaffold_10:4798123:4798253	0	0	212
A-m0070_3p	ACGAGTTTCCGGAGGCTGTTT	scaffold_18:3796259:3796581	0	0	38
A-m0059_5p	TTAGAGAGAGCAGAAAGAACA	scaffold_17:2570021:2570225	0	0	41
A-m0089_3p	TGTTTGTCAGTGTGGTTGCGGTT	scaffold_1:23370327:23370491	0	0	42
A-m0100_3p	TAATATGTGGATATGCCAGCGG	scaffold_2:22827035:22827254	0	0	46
A-m0107_3p*	TCGAATTTGGGCTTGAGATTG	scaffold_3:9383293:9383385	0	0	47
A-m0165_5p*	ACCAACCATTGACTTTGGCAGC	scaffold_8:366866:366938	0	0	74
A-m0108_3p	AGATTACGTTAGTTTCCTCTC	scaffold_3:15044403:15044569	0	0	88
A-m0122_3p	TCCGTTGTAGTCTAGTTGGT	scaffold_4:17294036:17294209	0	0	284
A-m0150_5p*	TGAAGAGGTAGAGAGTGTAATT	scaffold_6:26618407:26618570	0	0	297
A-m0126_3p#	TGTTTGGAAGTGTGGTAGCGGTT	scaffold_4:15905252:15905392	0	0	388

SD8: short day treatment for 8 weeks; C5: chilling treatment for 5 weeks; LD3: long day treatment for 3 weeks. *indicated a miRNA star (miRNA*) was observed; #indicated the expression of miRNA was confirmed by qRT-PCR.

**Figure 6:**
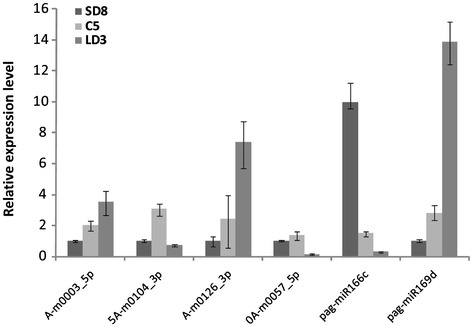
**The relative expression levels of known and novel miRNAs evaluated by qRT-PCR.** 5.8S rRNA was used as an endogenous reference. SD8: short day treatment for 8 weeks; C5: chilling treatment for 5 weeks; LD3: long day treatment for 3 weeks.

To validate the predicted novel miRNAs and confirm the expression profiles determined by Illumina high-throughput sequencing technology, we performed quantitative real-time PCR (qRT-PCR) on a subset of six miRNAs sequences, including two conserved and four novel miRNAs from SD8, C5 and LD3 (Figure 6). Most of the expression patterns were in agreement with our sequencing data, while a few miRNAs did not show the same expression trends. For example, the expression level of A-m0126_3p in C5 was measured to be higher by qRT-PCR than by the sequencing reads, which may be caused by a lack of sequence depth.

### Prediction of novel miRNA targets and RACE validation

A web-based miRNA target prediction program was employed to hunt for potential miRNA target genes. A total of 763 unigene sequences were predicted to be the targets of 119 novel miRNAs in SD8, 942 unigene sequences to be the targets of 107 novel miRNAs in C5 and 833 unigene sequences to be the targets of 126 novel miRNAs in LD3. The number of predicted targets varied from 1 to 34 per miRNA and most had three to seven targets. To focus on the biological processes, we predicted the targets of novel miRNAs that were specifically expressed in one phase or that had expression changes during the phase transition (Table 4). Although the target genes of some of these miRNAs showed distinct functions, a portion of them predicted the target as a single gene or members of a gene family. Many of these targets were involved in energy metabolism and solute transport, representing the dramatic metabolism changes between dormancy and active growth. Several targets were annotated as NBS resistance protein and leucine rich repeat protein, suggesting the direct response to adverse environment. Additional, some novel miRNAs targeted cell signaling-related genes, which could lead to expression change of these genes in the annual cycle.

**Table 4:** **Target prediction and annotation of novel miRNAs with marked expression change during dormancy-activity transition**

**miRNAs**	**Target genes**	**Annotations**
0A-m0034_5p	POPTR_0011s05660	Transcription factorPHOX2/ARIX
	POPTR_0007s07100	Ribonucleotide reductase, alpha subunit
	POPTR_0006s24000	Predicted mitochondrial carrier protein
	POPTR_0005s08950	Ribonucleotide reductase
0A-m0035_5p	No prediction	
0A-m0134_3p	POPTR_0019s02795	Calmodulin binding protein
	POPTR_0014s08840	Photosystem II CP47 chlorophyll protein
	POPTR_0011s12360	COP1-Interacting Protein 7
0A-m0013_3p	POPTR_0019s02795	Calmodulin binding protein
	POPTR_0014s08840	Photosystem II CP47 chlorophyll protein
	POPTR_0019s02910	NBS resistance protein
0A-m0051_5p	POPTR_0017s09870	Galactose oxidase/kelch repeat superfamily
	POPTR_0001s33900	Galactose oxidase/kelch repeat superfamily
0A-m0066_3p	POPTR_0019s02910	NBS resistance protein
	POPTR_0014s08840	Photosystem II CP47 chlorophyll protein
0A-m0062_3p	POPTR_0007s15090	Histone acetyltransferase
0A-m0050_3p	POPTR_0001s25740	Anthranilate synthase, alpha subunit 2
0A-m0146_5p	POPTR_0009s07980	no functional annotations
0A-m0078_5p	POPTR_0001s38870	Leucine rich repeat protein
	POPTR_0005s00880	Leucine rich repeat protein
0A-m0045_5p	POPTR_0003s11180	no functional annotations
	POPTR_0018s07190	BREVIS RADIX-like 4
0A-m0007_3p	POPTR_0017s04700	Cc-NBS-LRR resistance protein
	POPTR_0017s00570	Cc-NBS-LRR resistance protein
	POPTR_0006s28970	no functional annotations
	POPTR_0006s00970	no functional annotations
0A-m0004_5p	POPTR_0010s00460	no functional annotations
	POPTR_0004s00300	Protein of unknown function (DUF506)
	POPTR_0002s18590	Protein phosphatase 2C
0A-m0084_5p	POPTR_0005s06530	ABC transporter family protein
	POPTR_0007s06200	Pentatricopeptide repeat-containing protein
	POPTR_0007s07050	Zinc finger protein
0A-m0077_5p	POPTR_0013s11290	Ubiquitin-conjugating enzyme
0A-m0108_5p	POPTR_0005s09600	Similar to nucleolin
	POPTR_0009s09760	Plant basic secretory protein (BSP) family protein
	POPTR_0005s06140	Alcohol dehydrogenase
0A-m0114_3p	POPTR_0107s00260	S-locus glycoprotein family
	POPTR_0107s00240	S-locus glycoprotein family
	POPTR_0107s00270	S-locus glycoprotein family
	POPTR_0107s00230	S-locus glycoprotein family
0A-m0149_5p	POPTR_0003s07030	Plant invertase/pectin methylesterase inhibitor
0A-m0057_5p	POPTR_0007s07340	Peroxidase
	POPTR_0001s22740	lupus la ribonucleoprotein
	POPTR_0012s13820	Ca2+/calmodulin-dependent protein kinase
5A-m0010_5p	POPTR_0006s03300	bZIP transcription factor
	POPTR_1554s00200	bZIP transcription factor
5A-m0081_3p	POPTR_0002s15530	No apical meristem (NAM) protein
5A-m0104_3p	POPTR_0008s11280	6-phosphogluconate dehydrogenase
	POPTR_0006s11050	Protein tyrosine kinase
	POPTR_0016s14560	Protein tyrosine kinase
5A-m0027_3p	POPTR_0014s13420	Elongation factor Tu
	POPTR_0009s02980	Domain of unknown function (DUF966)
	POPTR_0016s04110	Light stress-regulated 1
A-m0003_3p	POPTR_0017s03190	no functional annotations
	POPTR_0017s03260	no functional annotations
A-m0070_3p	POPTR_0001s27600	Nuclear polyadenylated RNA binding protein
A-m0059_5p	POPTR_0016s14740	Zinc transporter and related ZIP domain-containing proteins
	POPTR_0006s11190	Zinc transporter and related ZIP domain-containing proteins
	POPTR_0002s14740	3-phosphoshikimate 1-carboxyvinyltransferase
A-m0089_3p	POPTR_0019s02910	NBS resistance protein
	POPTR_0019s02795	Calmodulin binding protein
A-m0100_3p	POPTR_0018s02600	Lysosomal Pro-X carboxypeptidase
A-m0107_3p	POPTR_0001s06660	Mitochondrial transcription termination factor
	POPTR_0003s18980	Mitochondrial transcription termination factor
	POPTR_0001s07200	Mitochondrial transcription termination factor
A-m0165_5p	POPTR_0046s00370	no functional annotations
	POPTR_0003s09790	NBS-lRR resistance protein
	POPTR_0001s35160	Domains rearranged methyltransferase 2
A-m0108_3p	POPTR_0003s15190	no functional annotations
	POPTR_1856s00200	no functional annotations
A-m0122_3p	POPTR_0011s11810	MATE efflux family protein
	POPTR_0131s00210	MATE efflux family protein
A-m0150_5p	POPTR_0001s18390	Sulfite exporter TauE/SafE family
	POPTR_0007s12960	3-methyladenine DNA glycosidase
A-m0126_3p	POPTR_0019s02795	Calmodulin binding protein
	POPTR_0019s02910	NBS resistance protein

**Figure 7:**
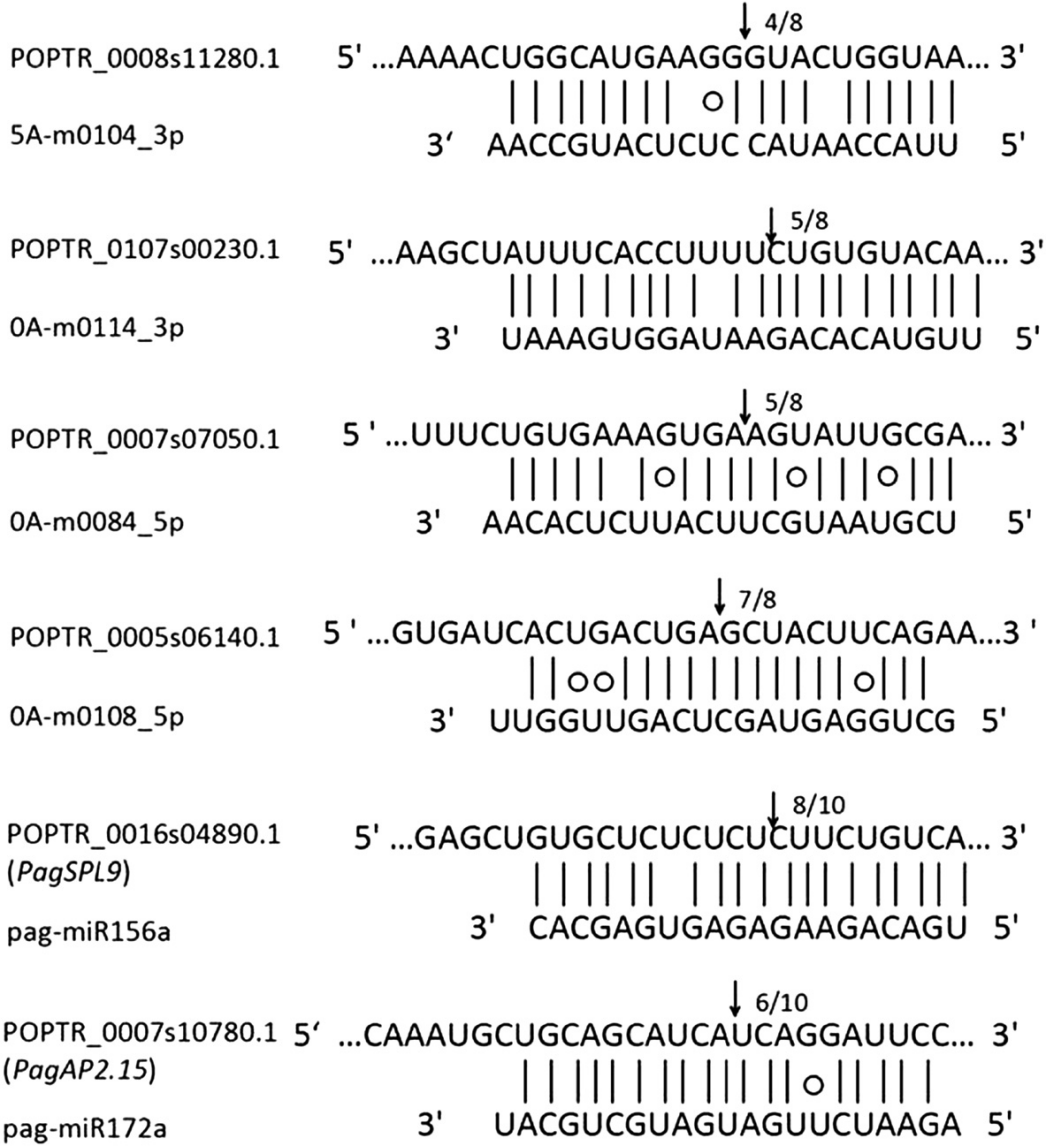
**RACE validation of known and novel miRNAs.** The recognition site of each target mRNA was aligned with corresponding miRNAs. The arrows indicated the cleavage sites of target genes, and the numbers showed the frequency of cloned RACE products.

To validate the cleavage events of novel and known miRNAs, a modified RNA ligase-mediated rapid amplification of cDNA ends (RLM-RACE) experiment was performed to verify the miRNA-guided mRNA cleavage events. We tested four novel miRNAs and two known miRNAs to verify their ability to cleave their targets. All of the cleavage sites were located between 10 and 11 nucleotides relative to the 5′end of the complementary miRNA sequence, which was the characterized cleavage site of almost all of the known miRNAs (Figure 7). The RACE products of miR156 and miR172 were cloned and sequenced. Their alignments to the poplar genome showed that the targets of miR156 and miR172 were homologs of the DNA-binding transcription factors *SQUAMOSA PROMOTER BINDING PROTEIN-LIKE* (*SPL*) and *APETALA2* (*AP2*), respectively, and the targets of other novel miRNAs were identical with the computer prediction in Table 4.

## Discussion

Plant miRNAs have a wide range of regulatory functions in many biological and metabolic processes, including developmental regulation, cell differentiation, signal transduction, growth control, and biotic and abiotic stresses [40]. Although an increasing number of poplar miRNAs have been identified in tissues or under certain environmental conditions [48], and some of them have been well characterized to involve various developmental process [35,49], little is known about the roles of miRNAs in the cambium dormancy regulation in trees. We have presented here a comprehensive analysis of sRNAs in the dormancy-active growth transition at the genome-wide level, which revealed dynamic features of sRNA populations in the annual growth cycle and expression patterns of miRNAs involved in this process. In addition, a set of novel miRNAs with notable expression pattern changes was identified. Together, these results provide novel insights into the regulatory mechanism of the dormancy-active growth transition mediated by miRNAs.

### Deep sequencing reveals a diverse set of sRNAs in the cambium of poplar

Using high-throughput sequencing technology, we obtained more than 3 million unique sRNAs reads from three cambium samples during the dormancy-active growth transition in poplar. Although sRNAs are complex in composition, the large majority are 21 nt and 24 nt in plants [19], and the proportion of miRNAs varies between different species and upon environmental conditions [22,50,51]. The 24 nt sRNAs were mainly composed of siRNAs associated with repeats and transposons [52]. In our case, the sRNA length distribution patterns diverged during the dormancy-active growth transition. In dormancy, including endodormancy and ecodormancy, the 21 nt long sRNAs constituted the most abundant class, while in active growth the 24 nt long sRNAs constituted the most abundant class. We determined the size distributions in previous studies in poplar, and found that the 21 nt sRNAs were the major component in leaves and vegetable buds [20,48], while the xylem tissue has a major peak at 24 nt [48], which was in agreement with our data during active growth. We also found that the proportion of total miRNAs in dormancy, including both endodormancy and ecodormancy, was greater than that in active growth, which confirmed the induction of the 21 nt miRNA by dormancy. The increase in 24 nt sRNAs during active growth suggested that the 24 nt sRNAs, which would be mainly siRNAs known to guide DNA methylation and heterochromatin formation [53], may participate in the regulation of cambium activity, including cell division, cell differentiation and phytohormone regulation. The reversal of 21 nt and 24 nt sRNA abundance in the dormancy-active growth transition also indicated that these two kinds of sRNAs may play different roles during the annual growth cycle.

### Unique expression patterns of miRNAs in dormancy-release in poplar

Hundreds of miRNAs have been surveyed in poplar since next generation sequencing technology has become widely used, but little is known about miRNAs in tree dormancy regulation, especially the transition between dormancy and active growth. In this study, we found a series of miRNAs that might be involved in this process. for instance, most of the developmental-related miRNAs, especially those involved in meristem activity or cell proliferation, presented specific expression patterns. In *Arabidopsis*, increasing evidence shows that miR164, miR319, miR396 and their targets form a miRNA regulatory network to regulate cell proliferation, leaf development and meristem activity [40,54,55]. Intriguingly, these three miRNAs showed similar expression pattern during the endodormancy release process. The increasing expression levels of these three miRNAs in active growth suggested that a miRNA network regulating cell proliferation also existed in active cambium cells. Considering that both cambium and the leaf primordium are capable of cell division, the high level of these three miRNAs in active growth is quite reasonable, and the cessation of cell division in dormancy may cause the low abundance of these three miRNAs. Another miRNA that is crucial for vascular development is miR166, which regulates the *class III HOMEODOMAIN-LEUCINE ZIPPER* family of transcription factors. The relationship between miR166 and its target is essential for leaf abaxial/adaxial polarity establishment [40,56]. Unlike other developmental-related miRNAs, miR166 was more abundant in dormancy, and had a very low expression level in active growth, which was in agreement with a previous study in poplar [29]. These results indicated that miR166 was down-regulated in active growth to increase the expression level of its target gene, which had an important role in vascular development. In addition, the original functions of these miRNAs were mainly found in the shoot apical meristem or leaves, thus the existence of these miRNAs in cambium suggested they may share the same regulatory mechanism in different tissues.

Interestingly, miR168 was found to be up-regulated in active cambium. The target of miR168 was *AGO1*, which was the key regulator of miRNA biogenesis [42]. The high expression level of miR168 leads to the repression of *AGO1*, which causes a reduction in the miRNA expression level. As expected, the total miRNAs in active growth decreased, indicating that miR168 was involved in the miRNA biogenesis as a feedback regulator in the cambium stage transition.

miR156 and miR172 target DNA-binding transcription factors *SPL* and *AP2* genes, respectively, which control the juvenile-to-adult vegetative transition both in annual herbs [57,58] and woody perennial plants [35]. They show converse expression patterns and regulatory relationships during the phase transition [57]. Surprisingly, the expression levels of miR156 and miR172 also had opposite expression patterns in our study. The 5′RACE results confirmed the cleavage events in miR156 and miR172, suggesting that the two miRNAs were functional during the dormancy-active growth transition. These similar expression patterns suggested the complementary of miR156 and miR172 might play an important role in this process, which needed to be experimentally confirmed.

### Auxin-related miRNAs may participate in the regulation of endodormancy release

A continuous chilling is the only natural way to release endodormancy and transition to ecodormancy. The main difference between endodormancy and ecodormancy is that ecodormant trees have the ability to respond to growth-promoting signals, such as auxin or appropriate outside conditions. In other words, the chilling triggers the ability of the tree to respond to auxin. In our data, most miRNAs had significant expression-level changes between dormancy and active growth, but only a few of them had changes between endodormancy and ecodormancy. Among them, miR160, whose target was *AUXIN RESPONSE FACTOR 10/16/17* (*ARF10*/*ARF16*/*ARF17*) [59-61], was highly expressed in ecodormancy. Lu *et al*. studied the cold-responsive miRNAs in poplar by microarray analysis, and showed that ptc-miR160a-g were strongly induced by cold treatment for 12 h, and that this induction disappeared after 16 h of treatment [46]. In our case, the chilling lasted 5 weeks, so this high abundance of miR160 in ecodormancy may not be due to cold tolerance, but ecodormancy itself. In *Arabidopsis*, miR160’s target genes are negative regulators of auxin signaling [62,63], so it is possible that the highly expressed miR160 may enhance the auxin signal by repressing its targets. The other two auxin-related miRNAs, miR390 and miR167, increased dramatically during active growth, suggesting that they are involved in the auxin signal pathway during active growth. The results showed that the miRNAs mediating the auxin signal pathway had complex regulatory roles in the cambium dormancy phase transition.

### Novel miRNAs and their putative targets during the dormancy-release in poplar

Hundreds of novel miRNAs as well as their targets were identified in this study. Some of them may have important roles in the dormancy-activity transition. For instance, 0A-m0062_3p which had a high expression level in active growth targeted a histone acetyltransferase gene. Considering the close link between histone acetylation and gene activation [64], this result suggested that some changes of gene expression in endodormancy could be caused by the regulation of the histone-modifying enzyme by miRNAs. Additional, one putative target of an active growth-specific novel miRNA, A-m0165_5p, was annotated as a homolog of *DOMAINS REARRANGED METHYLTRANSFERASE 2*, which catalyzes *de novo* methylation and is responsible for RNA-directed DNA methylation in *Arabidopsis* [65]. Together, these findings might suggest a miRNA-guided regulation of epigenetic modifications in dormancy and active growth. Among dormancy-highly-expression novel miRNAs, 0A-m0077_5p, which pairs with an ubiquitin-conjugating enzyme and 0A-m0149_5p, which matches a plant invertase/pectin methylesterase inhibitor, were also detected, suggesting different protein levels and cell wall components in annual cycle. Compared with the higher numbers in active growth and endodormancy, only four novel miRNAs with high expression levels in ecodormancy are listed in Table 4. Among them, 5A-m0010_5p targeted a homolog of the No Apical Meristem (NAM) protein, suggesting that this novel miRNA may participate in meristem activity. Interestingly, the target of 5A-m0104_3p with the highest expression in ecodormancy was RACE validated and annotated as a homolog of 6-phosphogluconate dehydrogenase, which is an oxidative carboxylase that catalyzes the decarboxylating reduction of 6-phosphogluconate into ribulose 5-phosphate in the presence of NADP. Since a previous study showed that the activity of 6-phosphogluconate dehydrogenase underwent a significant change in poplar xylem between winter and summer [66], the high expression level of 5A-m0104 in ecodormancy may contribute to the physiological change during the transition from endodormancy to active growth. These findings raised the possibility of a regulatory role for miRNAs in metabolism and cell signaling, as well as epigenetic changes between dormancy and active growth.

## Conclusions

In summary, a genome-wide sRNA profile of the cambial meristem was performed to present the miRNAs involved in the cambial dormancy-active growth transition. As a result, 123 known miRNAs, comprising 26 miRNAs families with obvious expression changes, were obtained, which included developmental-, phytohormone-, stress- and physiological-related miRNAs. In addition, 275 novel miRNAs expressed in the cambium were identified, and 34 of them displayed unique expression patterns during the dormancy-active growth transition process. The relative expression levels of four novel and two known miRNAs were also confirmed by qRT-PCR. We predicted the target genes of these novel miRNAs and experimentally validated some of them using 5′RACE. This revealed not only important known miRNAs, which may contribute to the regulation of the dormancy-active growth transition, but also novel miRNAs and their possible target genes, which would provide new insights into the regulatory mechanisms of the process in trees.

## Methods

### Plant material

Poplar (*Populus alba* × *Populus glandulosa* cv. “84 k”) plantlets were cultured on 1/2 Murashige and Skoog (MS) media with 20 g/L sucrose at 25°C under a LD photoperiod for a month. The induction of dormancy and resumption of growth in poplar was constructed as previously described with some modifications [36]: The plantlets were transferred into a greenhouse at 25°C under a LD photoperiod for at least 6 months until approximately 1 meter in height. Then, the healthy trees were transferred to a growth chamber for SD treatment, the apical buds were observed and the cambial cell layers of the 10^th^ internodes were counted in anatomical sections taken weekly during the process to evaluate the dormant state. After 8 weeks of the SD treatment, the dormant trees were treated with chilling (4°C) in an illumination incubator under the same conditions for 5 weeks. The chilling-treated trees were transferred to the LD condition at 25°C and the sprouting of dormant buds was inspected every week. Then, the trees released from dormancy grew in the LD condition at 25°C for another 3 weeks to achieve the active growth stage. At least 12 trees were used in every step of the process.

For the anatomical sections, the stems of the 10^th^ internodes were fixed in formalin/acetic acid/alcohol, dehydrated in a gradient of ethanol solutions, and finally embedded into Spurr’s resin according to the manufacturer’s description. Sectioning was performed using a Leica microtome, stained with 0.1% (w/v) toluidine blue O (Sigma, St. Louis, MO, USA), and observed under a Zeiss Axioskop 2 Plus microscope equipped with a computer-assisted digital camera.

### sRNA sequencing and bioinformatic analysis

The cambial zones of 12 plants were sampled in SD8, C5 and LD3. The Illumina sequencing of sRNAs was performed following a previously published protocol [67]. The plant materials from the cambial zones were carefully scraped and ground in liquid nitrogen, and total RNAs were immediately extracted using Trizol reagent (Invitrogen, Carlsbad, CA, USA), and then separated on a 15% denaturing polyacrylamide gel. The 18–26 nt long sRNAs were excised and recovered. 5′ and 3′adapters were ligated to the isolated sRNAs, which were sequentially reverse transcribed and amplified by PCR. The purified PCR products were sequenced using a Solexa 1G Genetic Analyzer (Illumina, USA) at the Beijing Genomics Institute (BGI), Shenzhen, China.

The sequenced raw data were transferred into clean reads after removing the contaminants, low-quantity reads and adapters, which were used for the size distribution. SOAP software [68] was employed to map the clean reads to the poplar genome (http://www.phytozome.net/poplar). GenBank (http://www.ncbi.nlm.nih.gov/genbank/), NCBI (http://www.ncbi.nlm.nih.gov) and Rfm (http://www.sanger.ac.uk/Software/Rfm/) databases were used to annotate the rRNA, scRNA, snoRNA, snRNA and tRNA in the sRNA library [69]. The clean reads mapped to the exons and introns of mRNA in the poplar genome were annotated as degraded sequences. Known miRNAs were identified by alignment to sequence in miRBase 18.0 (http://www.mirbase.org/) with no mismatches [70].

The remaining unannotated reads were used to predict novel miRNAs through the following methods: the MIREAP (http://sourceforge.net/projects/mireap/) software was employed to predict potentially novel miRNAs. First, the secondary structures of the sRNA precursors were predicted by the RNAfold web server [71] (http://rna.tbi.univie.ac.at/cgi-bin/RNAfold.cgi) with default parameters. Additionally, we used the essential criteria for screening the miRNA candidates according to Meyer *et al.* [72].

### Target prediction of novel miRNAs

The miRNA target candidates obtained through the MIREAP software were used to predict their target genes. The sequences of novel miRNAs were submitted to the psRNATarget server (http://bioinfo3.noble.org/psRNATarget/), which contains plant miRNAs to screen target genes from Phytozome v. 9.1 (http://www.phytozome.net/poplar/) with criteria described previously [73,74]. The predicted target genes were annotated by searching the Phytozome v. 9.1 and NCBI databases.

### miRNA expression analysis

To calculate the relative miRNA expression level and determine if there was a significant expression-level change, we used the log2-ratio and Scatter plot to compare the expression levels of miRNAs expressed in the three libraries based on previously established methods [75,76]. First, samples were normalized to 1 million, regardless of the total number of miRNAs in each sample. After normalization, if the expression level of a miRNA was 0, then it was revised to 0.01; if the expression level of a miRNA gene in all the libraries was less than 1, then this miRNA was removed because its expression level was too low. The normalized reads were used to calculate the fold change and p-value as follows:

Fold change = log2 (the normalized treatment reads/the normalized control reads), and

$$ p\left(x\Big|y\right)={\left(\frac{N_2}{N_1}\right)}^y\frac{\left(x+y\right)!}{x!y!{\left(1+\frac{N_2}{N_1}\right)}^{\left(x+y+1\right)}}\begin{array}{c}\hfill C\left(y\le {y}_{\min}\Big|x\right)={\displaystyle \sum_{y-0}^{y\le {y}_{\min }}p\left(y\Big|x\right)}\hfill \\ {}\hfill D\left(y\ge {y}_{\max}\Big|x\right)={\displaystyle \sum_{y\ge {y}_{\max}}^{\varphi }p\left(y\Big|x\right)}\hfill \end{array}, $$

where N1 is the total number of reads in the control sequencing library (SD8), N2 is the total number of reads in the treatment sequencing library, x is the number of reads for a miRNA in the control library and y is the number of reads for a miRNA in the treatment library. In this study, we used endodormancy (SD8) as the control. All calculations were performed on a BGI Bio-Cloud Computing platform (http://cloud.genomics.org.cn).

### qRT-PCR of miRNA expression

sRNAs were isolated from the cambial zone materials of 12 plants in LD3, SD8 and C5 using a miRNApure Mini Kit (CW Biotech, Beijing, China) following the manufacturer’s instructions. Then, the sRNA was polyadenylated by poly (A) polymerase, and first-strand cDNA was obtained from polyadenylated sRNAs using the miRNA cDNA Kit (CW Biotech, Beijing, China) following the manufacturer’s instructions. qRT-PCR was carried out as described: the SYBR Premix Ex Taq™ kit (TaKaRa Bio Inc., Japan) and an ABI 7500 Fast Real-time PCR machine (Applied Biosystems, Foster City, CA, USA) were used to complete the amplification, and the reaction procedure was set up according to the manufacturer’s protocol. Three replicates were performed for each sample with 5.8S rRNA as an internal reference [46], and we used the 2^-ΔΔCT^ relative quantification method to calculate relative changes in gene expression [77]. All the primers are listed in Additional file 3: Table S3.

### miRNA-mediated cleavage of mRNA

To identify cleavage sites in the target mRNAs, a modified RLM-RACE was performed using a GeneRacer Kit (Invitrogen, Carlsbad, CA, USA). All the steps followed the manufacturer’s description, except that the calf intestinal phosphatase treatment was omitted to maintain the cleaved transcripts. All the primers are listed in Additional file 3: Table S3.

### Availability of supporting data

The data sets supporting the results of this article are included within the article and its additional files.

## Additional files

Additional file 1: Table S1. All the known miRNAs identified in this study with significant expression changes. Additional file 2: Table S2. All the novel miRNAs predicted in SD8, C5, and LD3. Additional file 3: Table S3. All the primers used in this study.
